# Supplementary material for: Negative dielectric constant of water confined in nanosheets
Source: Nat Commun. 2019 Feb 20;10:850. doi: 10.1038/s41467-019-08789-8 (PMC6382890; doi:10.1038/s41467-019-08789-8)
Supplement: Supplementary file 1 — Supplementary Information [file 41467_2019_8789_MOESM1_ESM.pdf]

Supplementary information

Negative Dielectric Constant of Water Confined in Nanosheets

Sugahara et al.

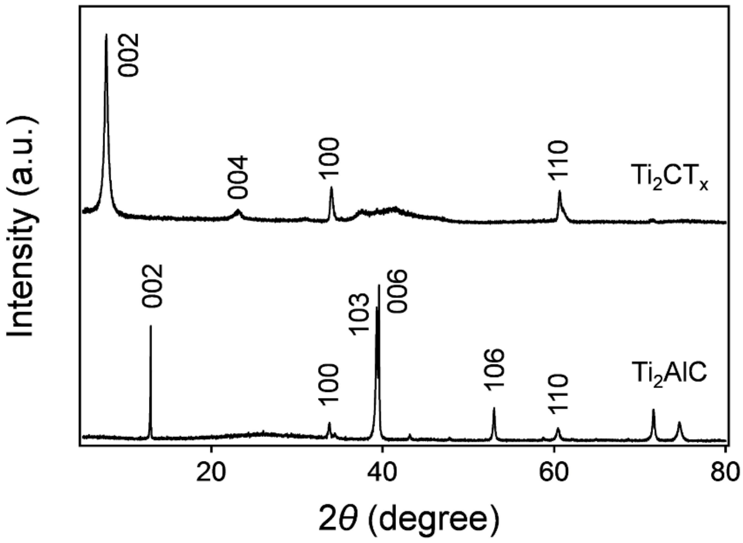

Supplementary Fig. 1. X-ray diffraction patterns for Ti_2AlC and anhydrous Ti_2CT_x (dried at 200 ºC). By treating Ti_2AlC with a HCl aqueous solution of LiF, Al layers were removed to afford MXene Ti_2CT_x, where the interlayer distance increased because of the attachment of surface termination groups (T).

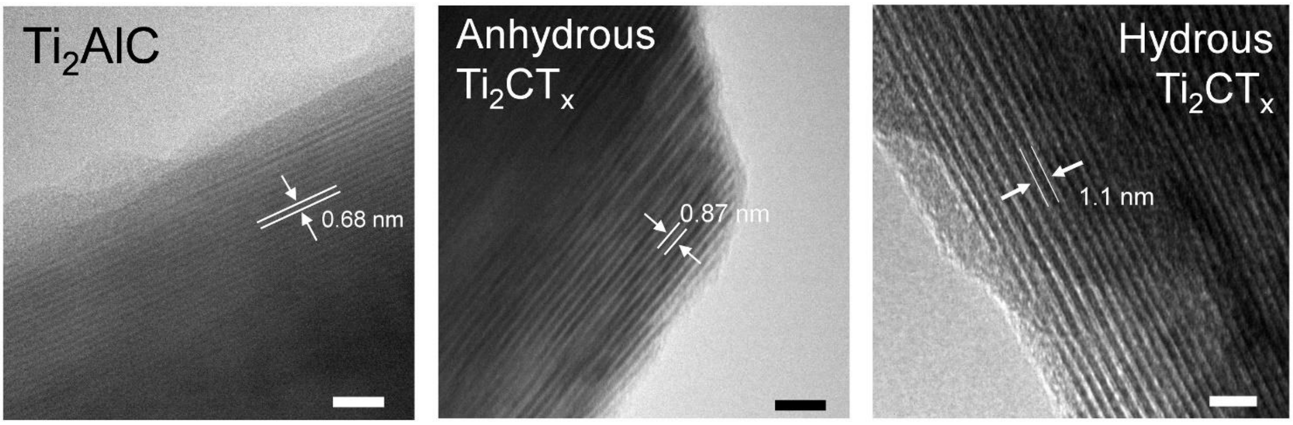

Supplementary Fig. 2. TEM images of Ti_2AlC, anhydrous Ti_2CT_x (dried at 200 ºC) and hydrous Ti_2CT_x (handled in ambient atmosphere). The two-dimensional nanosheets of Ti_2CT_x have a stacked structure. The scale bar is 5 nm.

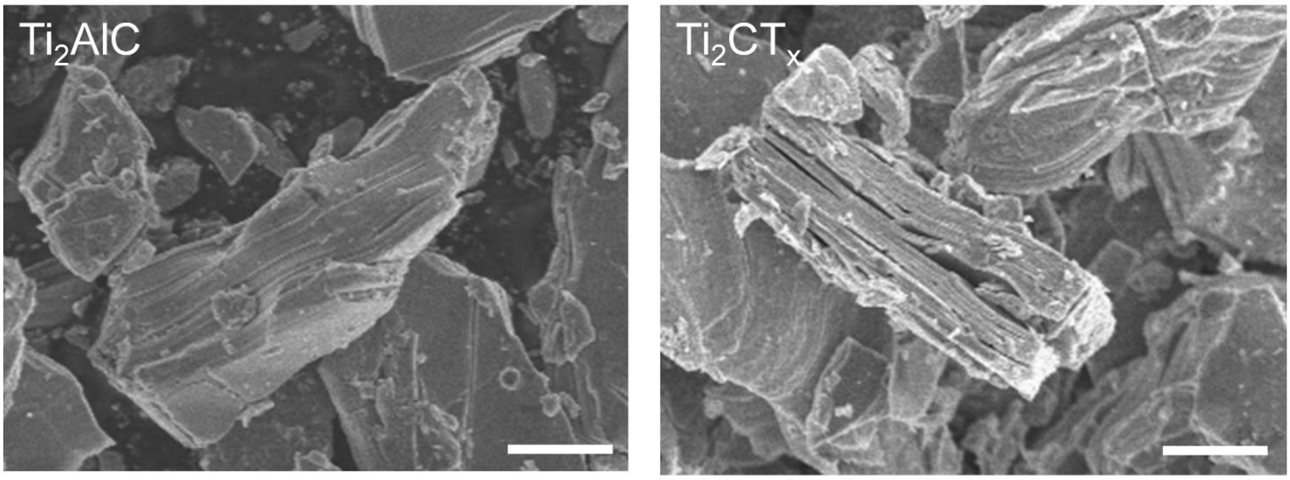

Supplementary Fig. 3. SEM images of Ti_2AlC and anhydrous Ti_2CT_x (dried at 200 ºC). Stacked Ti_2CT_x nanosheets partially have an exfoliated structure. The scale bar is 2 μm.

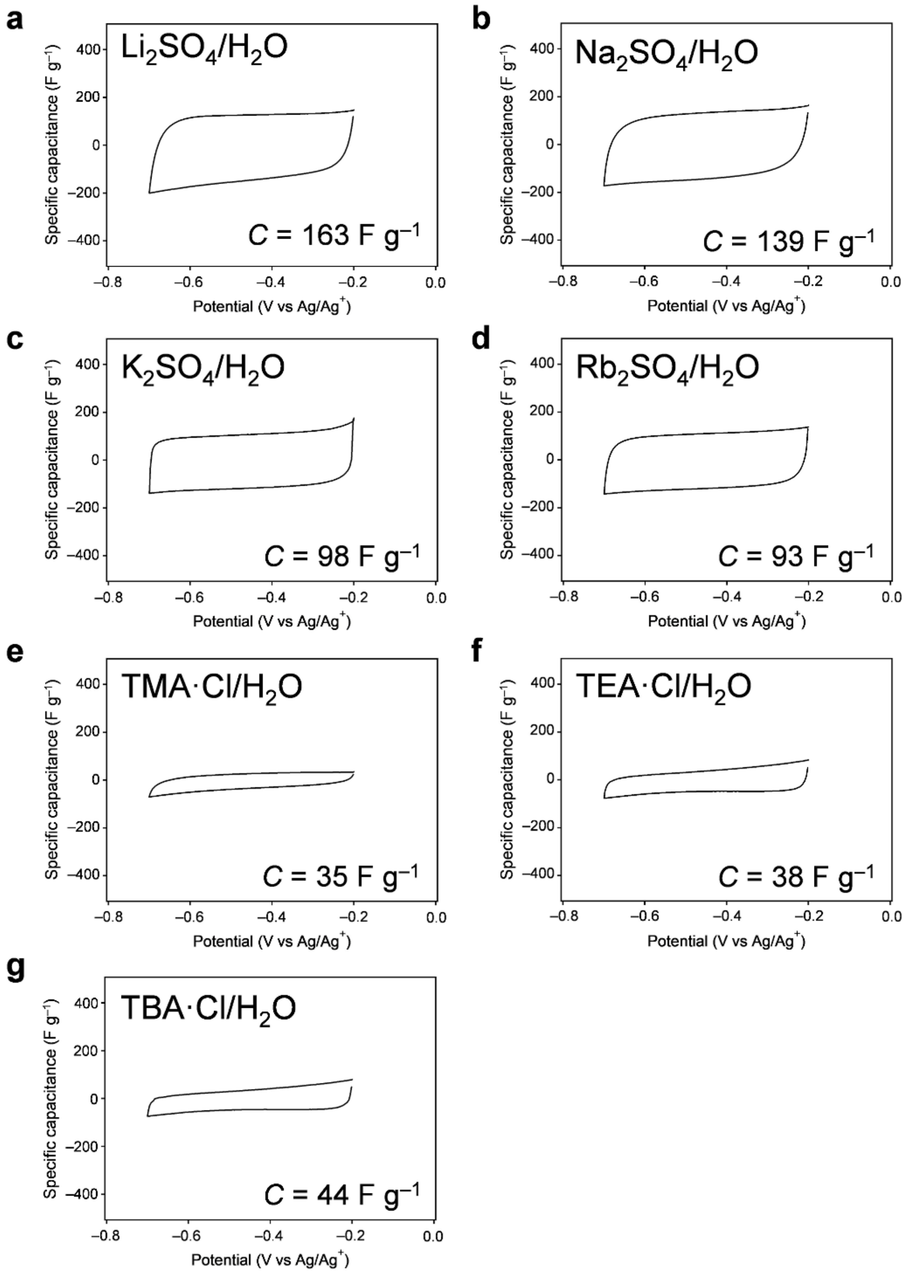

Supplementary Fig. 4. CV curves for Ti_2CT_x with various aqueous electrolytes at 0.5 mV s^{-1}. TMA, TEA, and TBA represent tetramethylammonium, tetraethylammonium, and tetrabutylammonium ions respectively.

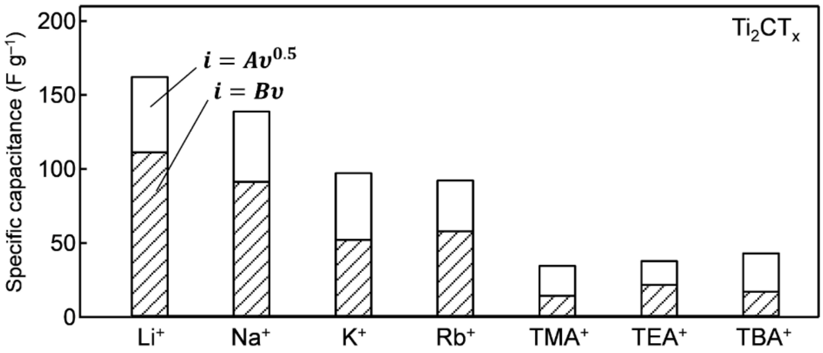

Supplementary Fig. 5. Surface and intercalation capacitances distinguished by the scan-rate dependence of CV. The surface and intercalation capacitances (hatched and white bars, respectively) were evaluated using the scan-rate (v) dependence of the capacitance ($i = Av^{0.5}+Bv$) from the CV curves of Ti_2CT_x with various aqueous electrolytes. However, the rate-dependent current i of MXene electrodes is governed by the Poisson-Nernst-Planck (PNP) equation under the initial and boundary conditions of a cylinder electrode. As this equation cannot be solved analytically, the widely-used rate dependence for diffusion current ($i = Av^{0.5}$) has no theoretical background.

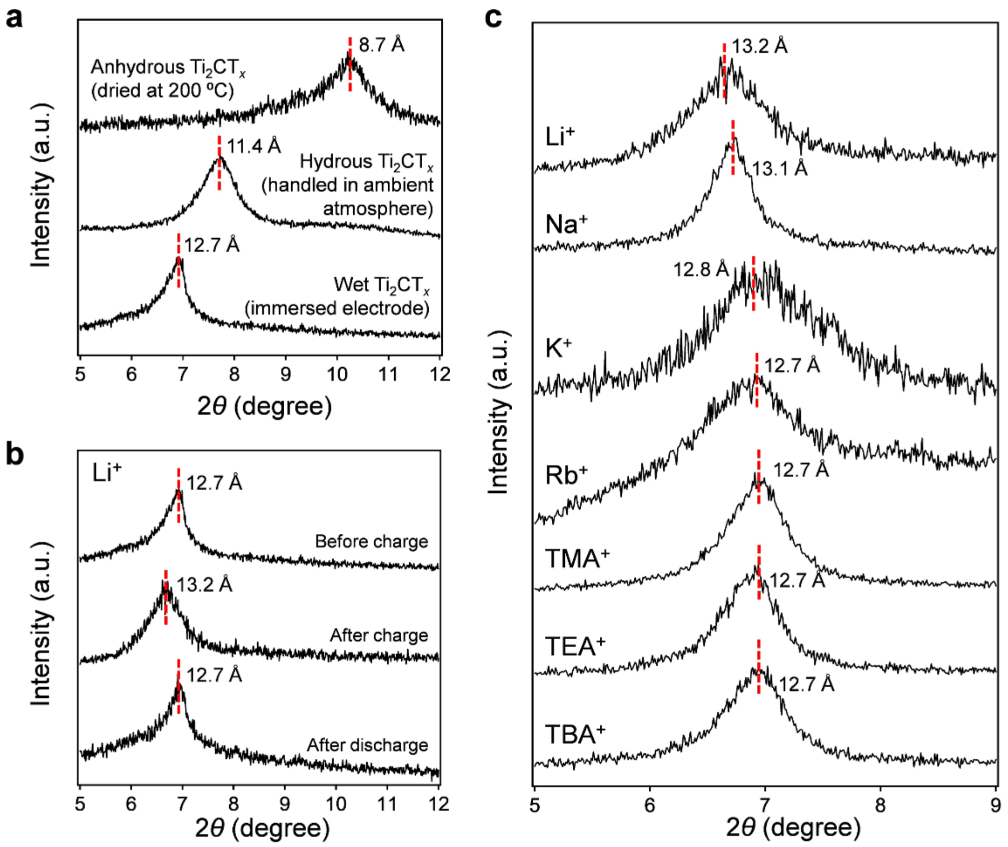

Supplementary Fig. 6. X-ray diffraction analysis of MXene electrodes. a, X-ray diffraction patterns for anhydrous Ti_2CT_x (dried at 200 ºC, handled in inert atmosphere), hydrous Ti_2CT_x (handled in ambient atmosphere), and wet Ti_2CT_x (electrode material immersed in an aqueous electrolyte). **b,** *Ex-situ* X-ray diffraction patterns for Ti_2CT_x upon charge/discharge with an aqueous Li^+ electrolyte. **c,** *Ex situ* X-ray diffraction patterns for Ti_2CT_x charged with various aqueous electrolytes. Assuming a Ti_2CT_x layer thickness of 8.7 Å based on the XRD pattern for anhydrous Ti_2CT_x, the separation in the slit walls (MXene surface) (2*b*) is calculated to be 4.5, 4.4, 4.1, and 4.0 Å for Li^+, Na^+, K^+, and Rb^+ intercalated MXene, respectively.

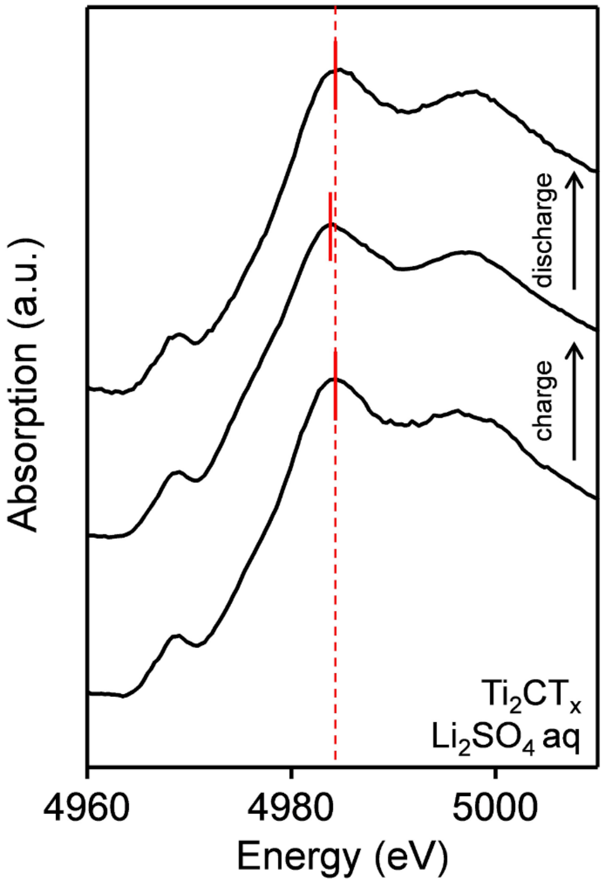

Supplementary Fig. 7. Ti *K*-edge X-ray absorption spectra for Ti_2CT_x in a Li_2SO_4 aqueous electrolyte. The reversible small shift of the main peak position upon charge/discharge indicates oxidation/reduction of Ti.

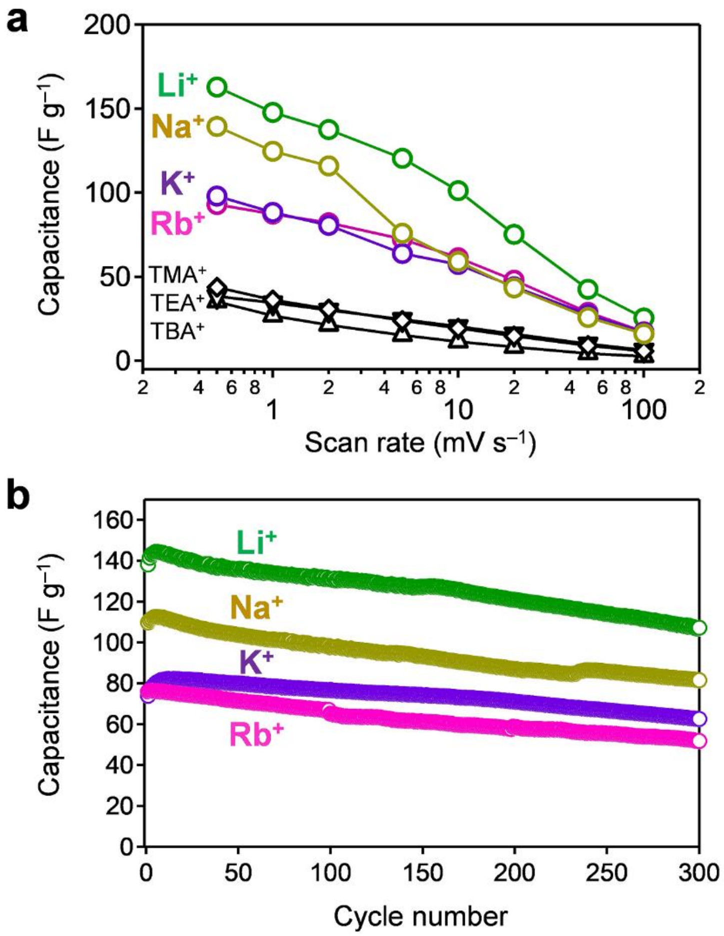

Supplementary Fig. 8. Electrochemical properties of Ti_2CT_x in aqueous electrolytes. a, Rate capability, and **b,** cycle stability from the CV measurements. The cycle stability was recorded at the scan rate of 2.0 mV s^{-1}. The Li^+ electrolyte gives the largest capacitance at each scan rate due to a negative dielectric constant of confined water. The enhanced capacitance is retained during 300 charge/discharge cycles.

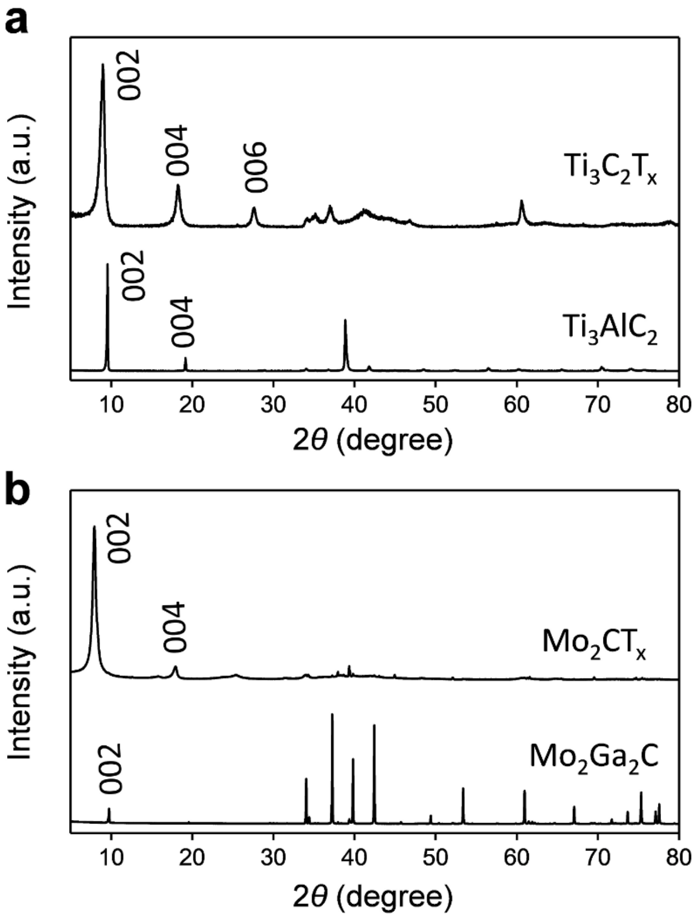

Supplementary Fig. 9. Powder X-ray diffraction patterns of MAX phases and MXenes. (a) Ti_3AlC_2 and $Ti_3C_2T_x$, (b) Mo_2Ga_2C and Mo_2CT_x. $Ti_3C_2T_x$ and Mo_2CT_x were synthesized by LiF/HCl treatment of Ti_3AlC_2 and Mo_2Ga_2C, respectively. For each case, the 002 diffraction shifts to a lower 2θ angle to indicate a typical increase of the interlayer distance after the LiF/HCl etching process.

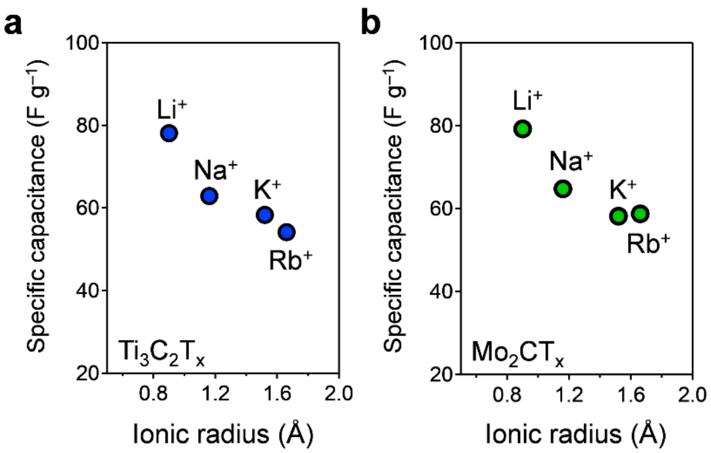

Supplementary Fig. 10. Capacitance enhancement in $Ti_3C_2T_x$ and Mo_2CT_x. Ionic radius dependence of the experimental specific capacitance of **a,** $Ti_3C_2T_x$ and **b,** Mo_2CT_x with aqueous Li^+, Na^+, K^+, and Rb^+ electrolytes. Each capacitance is calculated from the CV curve recorded at scan rate of 0.5 mV s^{-1}.

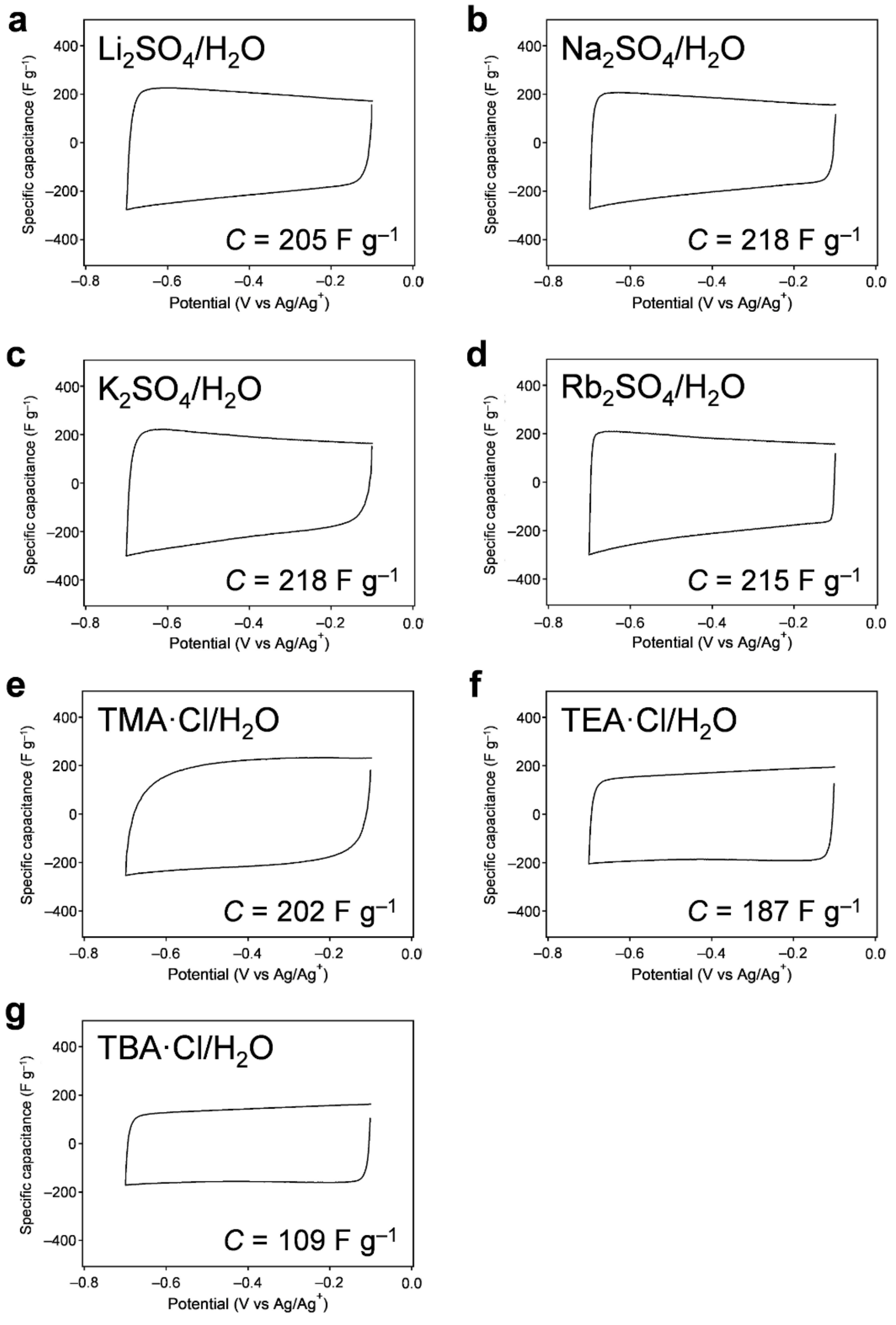

Supplementary Fig. 11. CV curves for conventional activated carbon EDLC electrodes at 0.5 mV/s with various aqueous electrolytes.

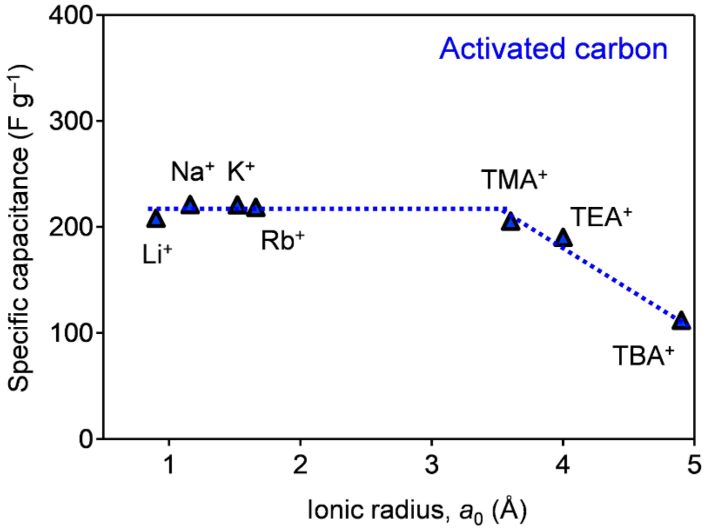

Supplementary Fig. 12. Specific capacitance of conventional activated carbon EDLC electrodes. Ionic radius dependence of the experimental specific capacitance of activated carbon with aqueous Li^+, Na^+, K^+, Rb^+, TMA^+ (tetramethylammonium), TEA^+ (tetraethylammonium), and TBA^+ (tetrabutylammonium) electrolytes. Each capacitance is calculated from the CV curve at the scan rate of 0.5 mV/s. The large tetraalkylammonium cation cannot diffuse into the micropore of activated carbon, leading to the smaller capacitance. On the other hand, hydrated alkali cations can diffuse into the micropore to give the constant capacitance.

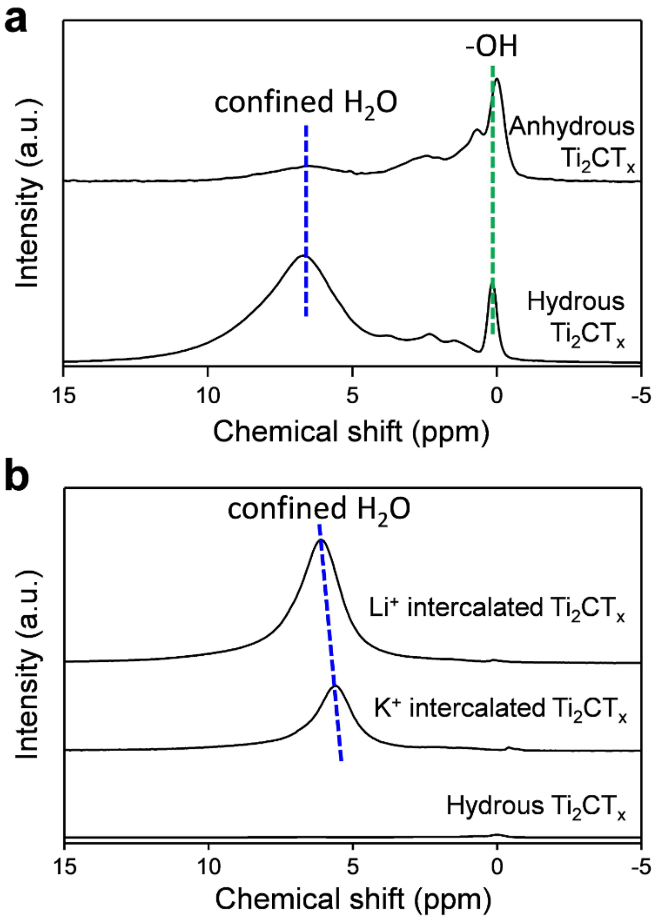

Supplementary Fig. 13. Water confinement in Ti_2CT_x. a, ^{1}H MAS NMR spectra for hydrous Ti_2CT_x and anhydrous Ti_2CT_x. Because the NMR peak at 7 ppm diminishes after the drying process, it can be ascribed to proton of confined water. The NMR peak at ca. 0 ppm may be ascribed to proton of -OH surface termination group. **b,** ^{1}H MAS NMR spectra for hydrous, K^+ intercalated, and Li^+ intercalated Ti_2CT_x. The spectra were normalized by the peak intensity at 0 ppm (proton of -OH surface termination group). The peak intensity at approximately 7 ppm increases after hydrated ion intercalation, which proves the existence of confined water. The peak intensity of the confined water after Li^+ intercalation is larger than that after K^+ intercalation, which confirms that the hydration energy of Li^+ is stronger than that of K^+. Interestingly, the chemical shift of the proton of confined water depends on intercalated alkali ions, presumably due to the difference in the hydration structure.

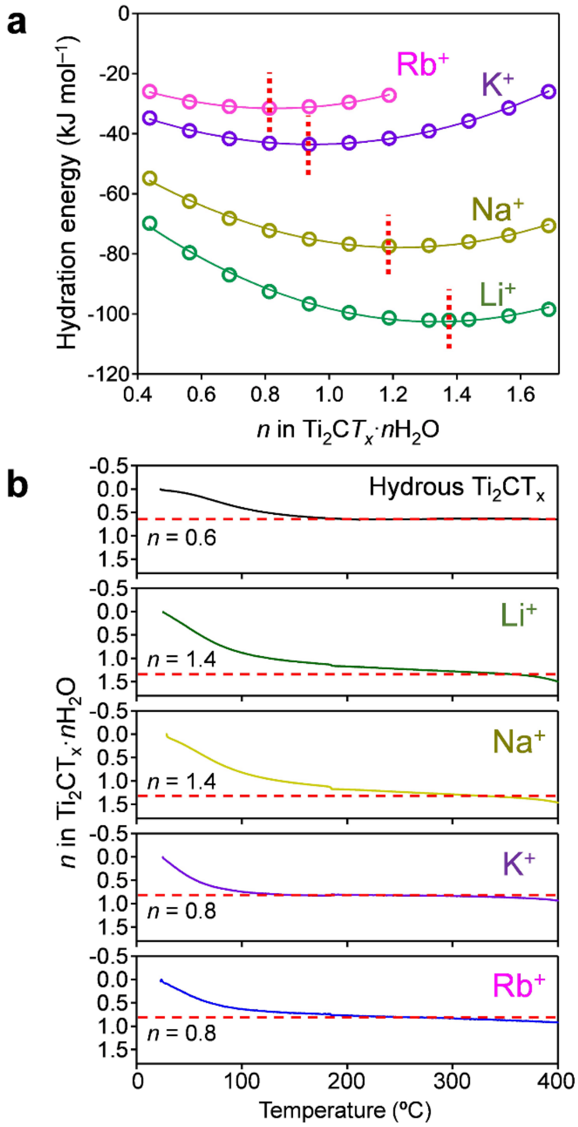

Supplementary Fig. 14. Theoretical and experimental determination of the number of water molecules (*n*) in MXene $Ti_2CT_x{\cdot}nH_2O$ electrodes. a, 3D-RISM calculated hydration energy of the $Ti_2CT_x{\cdot}nH_2O$ electrodes intercalated with various cations. Based on the electrochemical results, a model structure with the intercalation of 0.125 cation per formula unit (*e.g.*, $Li_{0.125}Ti_2CT_x{\cdot}nH_2O$) was used to calculate the hydration energy for each cation. **b,** Experimental thermogravimetric curves as a function of temperature for hydrous Ti_2CT_x (handled in ambient air), and Li^+, Na^+, K^+, and Rb^+ intercalated Ti_2CT_x electrodes. The temperature range is 30–400 °C using an Ar gas atmosphere. The heating rate was fixed at 5 K min^{-1}

a

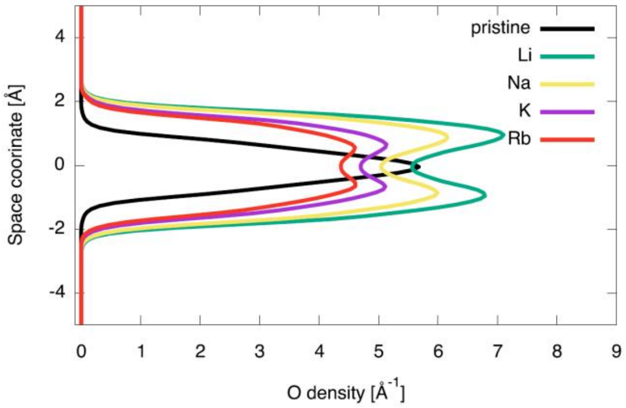

b

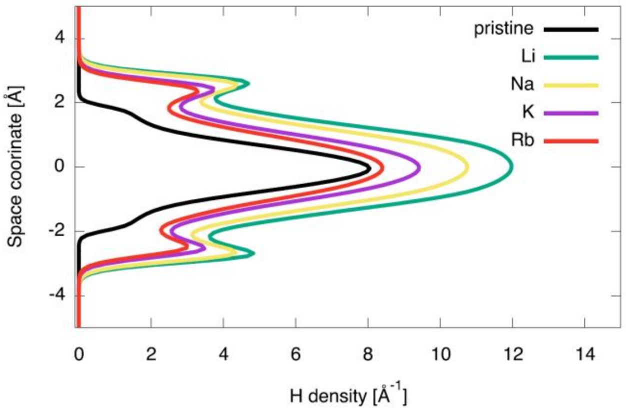

Supplementary Fig. 15. 3D-RISM calculated oxygen and hydrogen distributions. a, Calculated oxygen and **b,** hydrogen densities along the direction perpendicular to the MXene sheet intercalated with various cations.

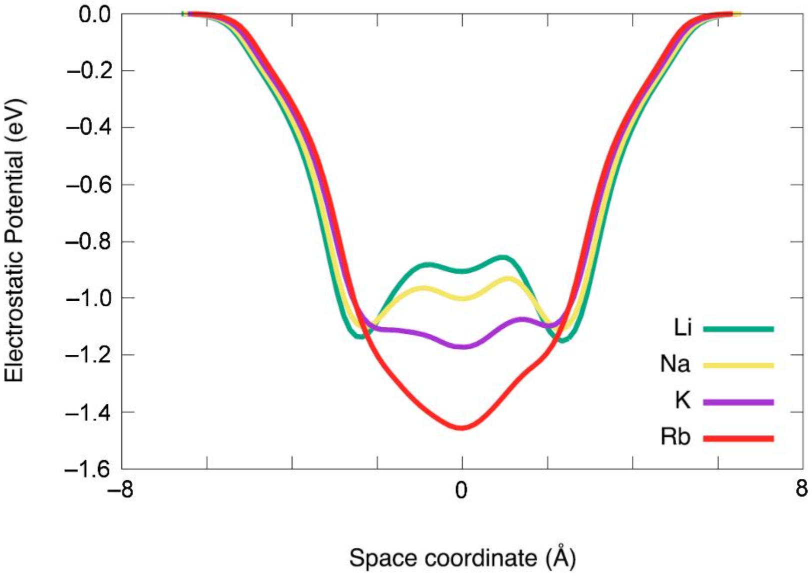

Supplementary Fig. 16. Electrostatic potential profile of Li^+, Na^+, K^+, and Rb^+ intercalated $Ti_2CT_x \cdot nH_2O$. Each cation locates at a space coordinate of 0 Å.

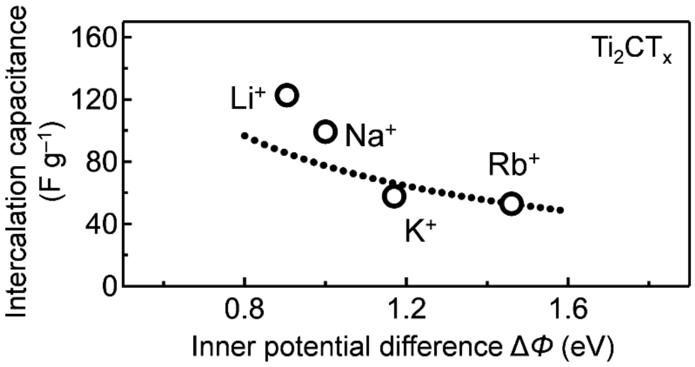

Supplementary Fig. 17. Intercalation capacitance of Ti_2CT_x with various aqueous electrolytes as a function of the inner potential difference calculated by 3D-RISM. The inner potential difference is related to the dielectric constant of water ($\lambda = \frac{l^{c}}{\varepsilon_{r}^{c}} + \frac{l^{h}}{\varepsilon_{r}^{h}}$). The broken line is the calculation result based on $C = \frac{\Delta Q_{\text{intercalation}}}{\Delta \Phi_{\text{calc}}}$, where $\Delta Q_{\text{intercalation}}$ = 80.6 C g^{-1} (0.125 cation intercalation per the formula unit).

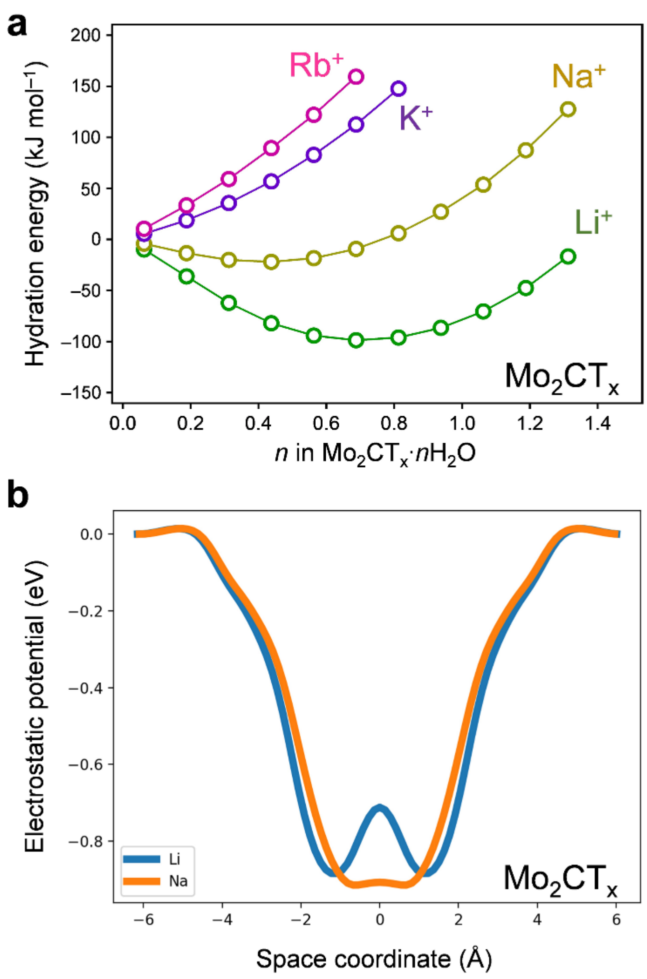

Supplementary Fig. 18. Negative dielectric constant of water confined in alkali-cation intercalated $Mo_2CT_x·nH_2O$. a, 3D-RISM calculated hydration energy of the $Mo_2CT_x·nH_2O$ electrodes intercalated with various cations. Based on the electrochemical results, a model structure with the intercalation of 0.125 cation per formula unit (*e.g.*, $Li_{0.125}Mo_2CT_x·nH_2O$) was used to calculate the hydration energy for each cation. While Rb^+ and K^+ intercalated MXenes do not exhibit hydration, Na^+ and Li^+ intercalated MXenes exhibit hydration. **b,** Electrostatic potential profile of alkali-cation intercalated $Mo_2CT_x·nH_2O$. Each cation locates at a space coordinate of 0 Å. Li^+ and Na^+ intercalated MXenes were calculated, because Rb^+ and K^+ in MXene do not have hydration shell that is expected to exhibit screening of the external electric field. The negative dielectric constant of a hydration shell is clearly suggested for Li^+.

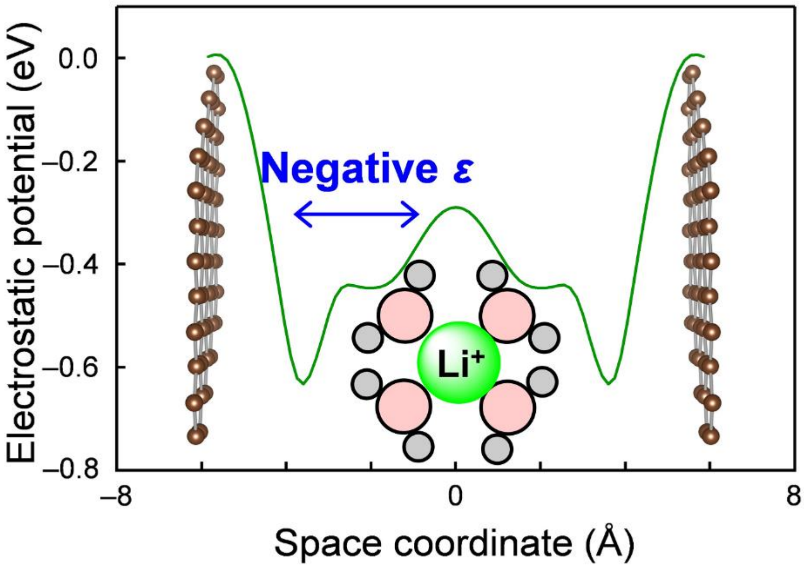

Supplementary Fig. 19. Electrostatic potential profile of Li^+ intercalated graphene. Li^+ locates at a space coordinate of 0 Å, while the separation of graphene sheets is assumed at 12 Å. After optimization of the water content for the fixed graphene-graphene distance, the electrostatic potential was calculated. Varying the separation shows that the negative dielectric constant of a hydration shell of Li^+ emerges approximately below 12 Å.

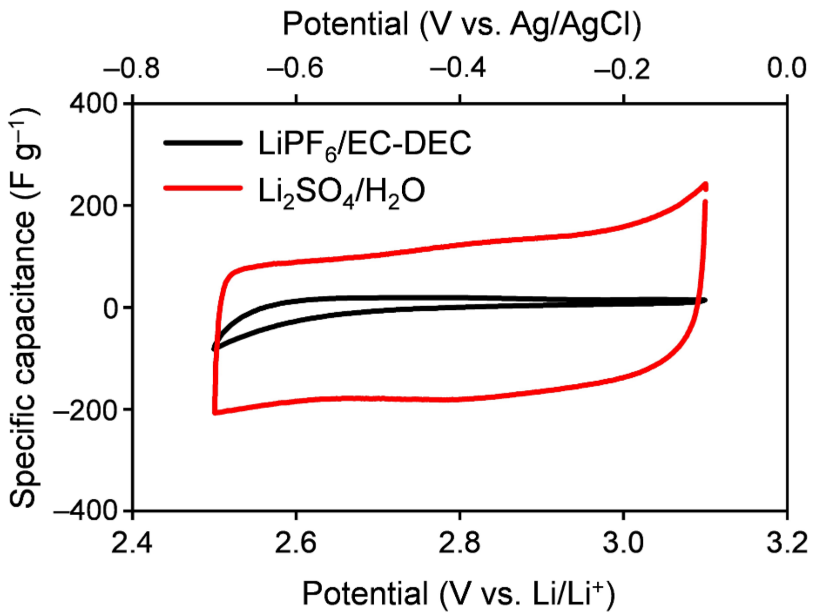

Supplementary Fig. 20. Comparison of CV curves of Ti_2CT_x in aqueous and organic Li^+ electrolytes at the scan rate of 0.5 mV s^{-1}. In the organic electrolyte, large solvated Li^+ cannot be intercalated maybe due to the large desolvation energy at the interface. However, other factors such as solvent cointercalation, or SEI formation should be considered to fully understand the capacitance with nonaqueous electrolytes, which will be reported elsewhere.

Plate	Cylinder		Slit	
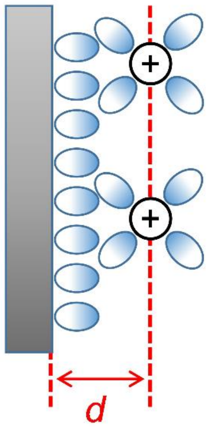	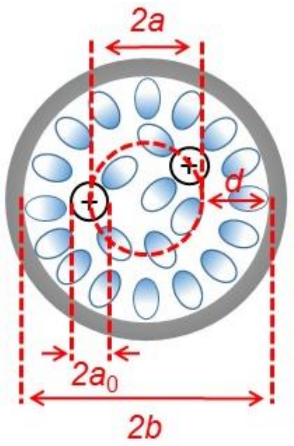		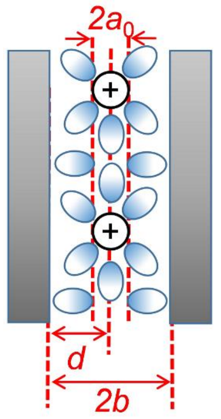	
$\frac{C}{A} = \frac{\varepsilon_r \varepsilon_0}{d}$	macrocylinder<br>$2b$ > 50 nm	$\frac{C}{A} = \frac{\varepsilon_r \varepsilon_0}{d}$	macroslit<br>$2b$ > 50 nm	$\frac{C}{A} = \frac{\varepsilon_r \varepsilon_0}{d}$ (macroslit and mesoslit)
	mesocylinder<br>$2b$ = 2–50 nm	$\frac{C}{A} = \frac{\varepsilon_r \varepsilon_0}{b\ln(b/(b-d))}$	mesoslit<br>$2b$ = 2–50 nm	
	microcylinder<br>2b < 2 nm	$\frac{C}{A} = \frac{\varepsilon_r \varepsilon_0}{b\ln(b/a_0)}$	microslit<br>$2b$ < 2 nm	$\frac{C}{A} = \frac{\varepsilon_r \varepsilon_0}{b-a_0}$

Supplementary Table S1. Schematic diagrams of parallel-plate, double-cylinder (wire-in-cylinder), and plate-in-slit capacitors. The corresponding capacitance equations are summarized below, where C is the capacitance, ε_r is the dielectric constant, ε_0 is the vacuum permittivity, and A is the surface area.

Supplementary Note 1. Charge storage mechanism in stacked MXenes

The specific capacitance (C) of a stacked MXene electrode is defined as $C = \frac{\Delta Q}{\Delta(\Phi_{cc} - \Phi_b)}$, where $\Delta(\Phi_{cc} - \Phi_b)$ is the change of the inner potential difference between a current collector (Φ_{cc}) and a bulk electrolyte (Φ_b), and ΔQ is a stored charge per the weight of the electrode (Supplementary Fig. 21). $\Delta(\Phi_{cc} - \Phi_b)$ is equivalent to the voltage change of an electrochemical cell when a reference electrode is used. $\Phi_{cc} - \Phi_b$ is expanded by the inner potential differences as,

$$\Phi_{cc} - \Phi_b = (\Phi_{cc} - \Phi_e^E) + (\Phi_e^E - \Phi_i^E) + (\Phi_i^E - \Phi_b).$$

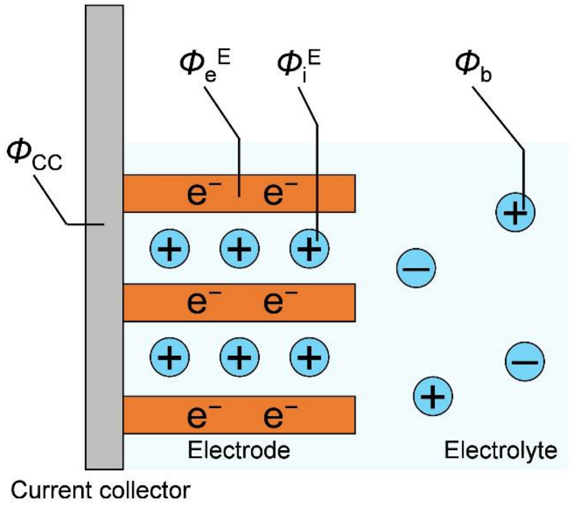

Supplementary Fig. 21. Inner potential distribution in charged MXene

The equilibrium condition of the electron electrochemical potential $\bar{\mu}_e$ and the ion electrochemical potential $\bar{\mu}_i$ between the current collector and the electrode material (MXene) respectively applies to give,

$$\mu_e^{cc} - F\Phi_{cc} = \mu_e^E - F\Phi_e^E, \text{ and } \mu_i^E + F\Phi_i^E = \mu_i^b + F\Phi_b.$$

Therefore,

$$\Phi_{cc} - \Phi_b = \frac{\mu_e^{cc} - \mu_e^E}{F} + (\Phi_e^E - \Phi_i^E) + \frac{\mu_i^b - \mu_i^E}{F}$$

Because μ_e^{cc} and μ_i^b is constant regardless of the state-of-charge of the electrode, $\Delta(\Phi_{CC} - \Phi_b)$ is expressed as,

$$\Delta(\Phi_{cc} - \Phi_b) = \Delta(\Phi_e^E - \Phi_i^E) - \frac{\Delta(\mu_e^E + \mu_i^E)}{F}$$

The density of states of the electrode is significantly large relative to the amount of the stored electron for aqueous electrolytes, thus μ_e^E is almost constant during the charge/discharge processes to give $\Delta\mu_e^E = 0$. In addition, because the number of the site for cation storage in the electrode is also significantly large relative to the amount of the stored cation, μ_i^E is almost constant during the charge/discharge processes to give $\Delta\mu_i^E = 0$. Therefore, $\Delta(\Phi_{cc} - \Phi_b) = \Delta(\Phi_e^E - \Phi_i^E) = \Delta\Phi$ applies to give $C = \frac{\Delta Q}{\Delta\Phi}$, and the electrode behaves as an electric double-layer (EDL) capacitor.
